# Integrating Statistical Machine Learning in a Semantic Sensor Web for Proactive Monitoring and Control

**DOI:** 10.3390/s17040807

**Published:** 2017-04-09

**Authors:** Jude Adekunle Adeleke, Deshendran Moodley, Gavin Rens, Aderemi Oluyinka Adewumi

**Affiliations:** 1 School of Mathematics, Statistics and Computer Science, University of KwaZulu-Natal, Westville Campus, University Road, Durban 3629, South Africa; gavinrens@gmail.com (G.R.); adewumia@ukzn.ac.za (A.O.A.); 2 CSIR Meraka Centre for Artificial Intelligence Research (CAIR), Meiring Naude Road, Brummeria, Pretoria 0001, South Africa; deshen@cs.uct.ac.za; 3 Department of Computer Science, University of Cape Town, 18 University Avenue, Rondebosch 7701, South Africa; 4 National Space Research and Development Agency, Obasanjo Space Centre, Airport Road, Abuja 900107, Nigeria

**Keywords:** proactive, Semantic Sensor Web, stream reasoning, situation prediction, machine learning, sliding window, multilayer perceptron

## Abstract

Proactive monitoring and control of our natural and built environments is important in various application scenarios. Semantic Sensor Web technologies have been well researched and used for environmental monitoring applications to expose sensor data for analysis in order to provide responsive actions in situations of interest. While these applications provide quick response to situations, to minimize their unwanted effects, research efforts are still necessary to provide techniques that can anticipate the future to support proactive control, such that unwanted situations can be averted altogether. This study integrates a statistical machine learning based predictive model in a Semantic Sensor Web using stream reasoning. The approach is evaluated in an indoor air quality monitoring case study. A sliding window approach that employs the Multilayer Perceptron model to predict short term PM${}_{2.5}$ pollution situations is integrated into the proactive monitoring and control framework. Results show that the proposed approach can effectively predict short term PM${}_{2.5}$ pollution situations: precision of up to 0.86 and sensitivity of up to 0.85 is achieved over half hour prediction horizons, making it possible for the system to warn occupants or even to autonomously avert the predicted pollution situations within the context of Semantic Sensor Web.

## 1. Introduction

Proactive monitoring of the natural and built environments is important in many day to day application scenarios in order to take control of environmental situations. Such application areas include preventing natural disasters, avoiding life threatening situations, enhancing productivity and improving health and well-being. For example, monitoring and control of indoor air quality in homes where there are pregnant mothers and infants is essential to avoid excessive exposure of these sensitive and vulnerable groups to indoor pollutants [1]. Advancements in sensor technology has made available low cost sensors that are embedded in everyday devices capable of observing and monitoring different properties of the environment. The goal of proactive computing is to bridge the gap between the virtual and the physical world by making sensor equipped computing devices understand the environment, anticipate the user’s goal and act on his or her behalf [2,3].

Sensor Web Enablement (SWE), an initiative of the Open Geospatial Consortium, has provided standards and techniques for the discovery of sensors and their observations, exchange and processing of sensor observations, and tasking of sensors and sensor systems as services on the web [4]. In a typical sensor application, real world occurrences are measured and captured as observations by sensors, formatted and transmitted continually through a communication network to a processing device that analyses and makes sense of the streaming sensor observation data to determine if any responsive actions are required. SWE supports exposing sensor observation data on the web for a plethora of application areas. However, to provide more expressive descriptions and enhanced access to sensor data on the web, the Semantic Sensor Web (SSW) initiative aims to extend SWE with Semantic Web technologies [5].

Semantic Web technologies, for example, ontologies, can be used to model concepts and relationships in a domain of interest [6,7]. Standardization efforts in the SSW has led to the Semantic Sensor Network (SSN) ontology which has become the de-facto ontology for SSW applications [8]. Raw sensor observation data is annotated and encoded with semantic metadata which allows for the integration and fusion of sensor data from heterogeneous sources. It also facilitates reasoning to make inferences about the observed feature of interest in the environment by evaluating semantic queries on semantically enriched data [5,9]. However, the inherent nature of sensor data streams requires specialized techniques for analysis, to infer knowledge from the streaming observation data [9,10].

Stream reasoning is a growing multidisciplinary research area that extends Semantic Web techniques to streaming data on the SSW [11,12,13]. SSW techniques have been investigated for monitoring and providing environmental decision support in different application domains [14,15]. While some progress has been made in terms of classifying current situations of interest from streaming data and decision support to mitigate these situations, predicting future situations for proactive control remains a challenge. In this work, the term *situation* is understood as an interpretation of sensor data in the application domain [16] and used in the context of the states of monitored features in a physical environment. Hence, *situation analysis* encompass the process of detecting *(situation detection)* and predicting *(situation prediction)* a situation of interest. The challenge of a proactive application on semantically enriched streaming data includes analyzing the data to detect situations of interest, to anticipate future occurrences of the situations, to process and enact decisions, and for knowledge management.

Anticipating the future occurrence of a situation of interest in order to enhance or reduce its probability of occurrence in favor of the user is the goal of a proactive system [2]. Some recent efforts have proposed semantic methods for predicting knowledge in semantically annotated data streams [10,17,18], and although it is an active research area, it is young with new techniques still emerging. Statistical machine learning provides advanced techniques which support applying learning algorithms to learn certain properties and patterns of data to predict future trends. This study suggests that integrating *predictive machine learning algorithms* in a SSW monitoring system will allow for taking proactive control actions to enhance or avoid specific future situations in many environmental monitoring application areas, for example, *indoor air quality*.

Indoor air quality for occupational health is a growing concern [19,20,21] and a research area, where proactive monitoring and control in the SSW can be applied. Most research efforts in indoor air quality have been directed to monitoring concentration levels of indoor pollutants and exposure levels of individuals to the pollutants with applications that react to change in target situations [1,14,21]. Such applications allow for responsive actions to situations which have already occurred and are useful for minimizing the effect of these situations. Identifying a possible situation before its occurrence will allow for proactive actions to be taken to avert or enhance its occurrence. A proactive monitoring system in a home can anticipate trends of future pollution levels and trigger control actions to avert the occurrence of such a situation altogether and prevent occupants from exposure to unhealthy levels of pollution. Some recent research efforts have proposed achieving proactive behaviors by integrating predictions in context aware systems [22,23,24].

In a previous work [1], an ontology driven system for proactive indoor air quality monitoring and control was proposed. A domain ontology for indoor air quality that imports and extends the SSN ontology was modeled to support the system. The ontology-driven system was able to successfully detect air quality states *(situation detection)* from semantically annotated sensor data. When unhealthy situations were detected, the system was also able to alert the occupants and infer appropriate control actions to abate the situation by reasoning on the ontology. Essentially, the system in the previous work [1] was able to monitor and only react to situations that have already occurred *(reactive)*.

The main contribution of this research study is two-fold. The first is the exploration of machine learning for situation prediction from streaming indoor air quality sensor data. This resulted in the selection of a Multilayer Perceptron (MLP) model using a sliding window over the incoming air quality data to predict future values of PM${}_{2.5}$ pollution levels. Secondly, a mechanism for incorporating machine learning models in Semantic Sensor Web architectures to support situation prediction is proposed. This is to support taking appropriate control actions ahead of time in order to prevent the occurrence of a future unhealthy situation *(proactive)*. The approach is aimed at combining the high accuracy and performance of statistical predictive techniques and the expressiveness of semantic analytic techniques for proactive monitoring and control applications.

This paper is organized as follows. In Section 2, we present an overview of the proactive monitoring and control framework, and in Section 3 we evaluate the framework with an indoor air quality case study. Section 4 presents the experiments performed to choose appropriate machine learning method for modeling situation prediction in the case study scenario, while the integration of the predictive model into a stream reasoning framework is presented in Section 5. The system is evaluated in Section 6. Section 7 compares this work with related work and Section 8 presents a discussion, conclusion and future work.

## 2. Proactive Monitoring and Control in SSW

### 2.1. Conceptual Framework

**Figure 1:**
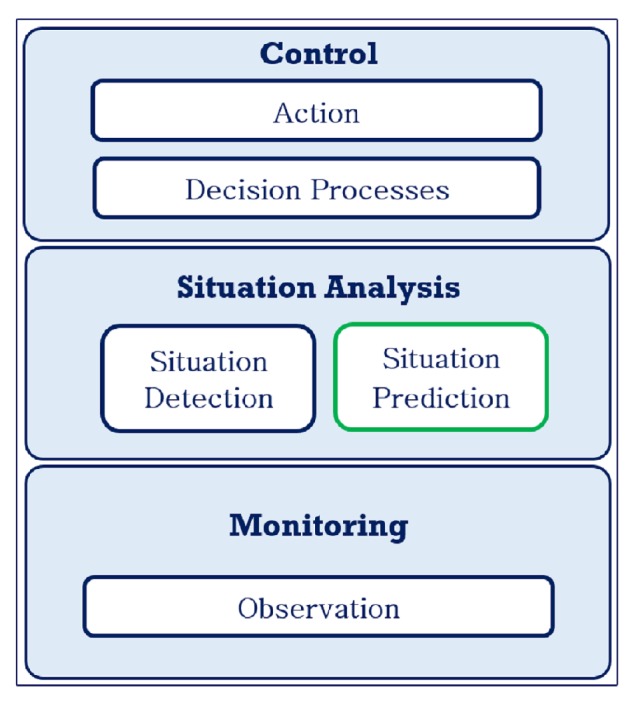
Conceptual model of proposed system.

The conceptual framework described in this section is an extension of our framework introduced in [1] (see Figure 1). The main extension is the incorporation of a situation prediction component in the situation analysis layer. Figure 1, shows the extended conceptual framework and indicates the situation prediction component which is the focus of this paper. The conceptual framework emphasizes the use of historical data to predict future situations. Statistical models can learn from historical data and use the weights generated to analyze current data to predict the future with potentially high precision and sensitivity.

The conceptual framework consists of three layers, which are discussed below:

- **Monitoring:** It serves as the interface between the system and the monitored environment where sensor observation data on the features of interest are captured. It represents certain parts of the system and ontology module that support data and measurements, including both the streaming sensor observation data and pre-captured static data in the system.
- **Situation Analysis:** It represents parts of the system and the ontology module that support situation detection and situation prediction, the two processes that generate the current and future states, respectfully. The integration of statistical predictive models take place in this layer and this forms the basis for the proactive behavior of the system. Situation analysis consists of two sub layers:
  – *Situation Detection:* this sub-layer supports the detection of situations of interest in the system based on defined indices, thereby identifying current states.
  – *Situation Prediction:* this sub-layer represents the part of the system which enables the prediction of the future states.
- **Control:** This layer consists of two sub layers that use the predictions to create decisions and that transforms the decisions into actions that can be carried out by either human or computer agents.
  – *Decision Processes:* This sub-layer represents parts of the system that are involved in deciding the control action to take, given the predicted future states. This layer fuses the identified current situation with the predicted situation to evaluate the most appropriate course of action.
  – *Action:* This sub-layer represents parts of the system that are used to enact the selected control action that corresponds to the result of the decision process.

### 2.2. Main Components

**Figure 2:**
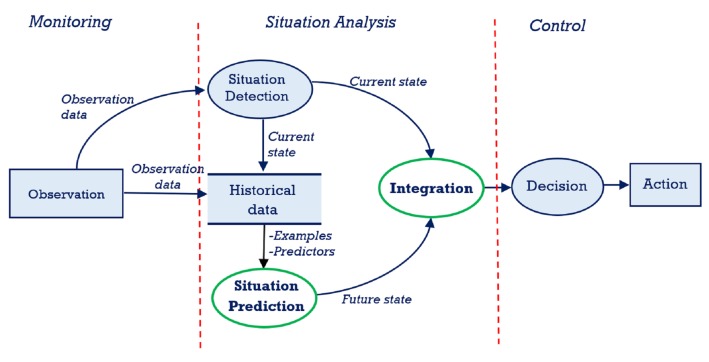
Dataflow diagram of the main components of the proactive architecture.

Figure 2 below shows the data flow through the main components of the system. Streaming data for proactive monitoring requires processing on the fly, the output of a process is automatically channeled as input for the next process. Historical data can be stored for later use, and temporary storage can be used to structure data as input for the next process. The observation data is streamed into situation detection and situation prediction components. The output of these two are integrated for decision processes. The decision output is then used by the action component to produce sets of actions to be performed by human or computer agents. The monitoring layer and situation detection component of the situation analysis layer has been reported in previous research [1]. The focus of this paper is on the implementation of the situation prediction process with a statistical machine learning based model and integrating the outputs of the situation analysis layer for the decision component. A detailed explanation of how we implemented the situation analysis component for the present study is given in Section 5. First, however, a case study and experiments are presented in order to motivate our approach for the implementation.

## 3. Application Use Case

The use case for this work is an ongoing cohort study [25,26] along with occupational health researchers investigating the effects of indoor air pollution, especially fine particles pollution on pregnant mothers and children. Particulate Matter, especially those of the aerodynamic diameter of 2.5 µm or less (also referred to as PM${}_{2.5}$), is one of increasingly incriminated indoor pollutants causing life threatening illnesses.

Predicting indoor pollution levels of PM${}_{2.5}$ in an indoor environment is a complex and challenging task. The indoor environment is a dynamic and complex system of various environmental phenomena, building features, human activities and infiltrations from the outdoor environment, all of which impact on the fine particles concentration. The proactive monitoring and control system is to predict PM${}_{2.5}$ pollution trends effectively and provide proactive control actions for the occupants when necessary to avoid excessive exposure to PM${}_{2.5}$ pollution.

### 3.1. The Area

The use case area is South Durban, a low income residential community in South Africa. The peculiar characteristics of housing in this community include lack of mechanical heating, ventilation or cooling systems, highly aggravated indoor pollutants through external pollution, and life style choices such as smoking and fossil fuel burning. The area is also in proximity of heavy industries; harmful effects of indoor pollution from outdoor sources have been noted to be more pronounced in residences that are close to heavy industries.

For this use case, the goal of the occupational health researcher is to keep the occupants’ exposure to particulate pollution within healthy limits. The World Health Organization (WHO) has recommended an exposure limit of 25 μg/m${}^{3}$ daily average for indoor environments [27,28]. Hence, a Proactive Pollution Monitoring and Control System (PPMC) is required to monitor and provide control actions to the residents when necessary to avoid exposure to unhealthy PM${}_{2.5}$ pollution levels. The system will predict the short term future trend of PM${}_{2.5}$ pollution and decide on appropriate control actions to stimulate proactive actions by the occupants to avert exposure to any anticipated unhealthy indoor PM${}_{2.5}$ pollution level. The indoor pollution will be controlled via the control of activities of occupants that influence indoor PM${}_{2.5}$ pollution. The control action will be communicated as a short message service (SMS) to advise the occupants on proactive actions to take in order to prevent the predicted pollution from occurring. This is an upgrade to the previous system [1], which only alerts the occupants of detected unhealthy situation that has already occurred.

Three different houses in the use case area were selected and used for testing the proactive monitoring and control system. One of the locations was first used as a pilot study for a week in April 2015, during the autumn season and the other two were used in October 2015, during the spring season.

### 3.2. The Proactive Pollution Monitoring and Control System

**Figure 3:**
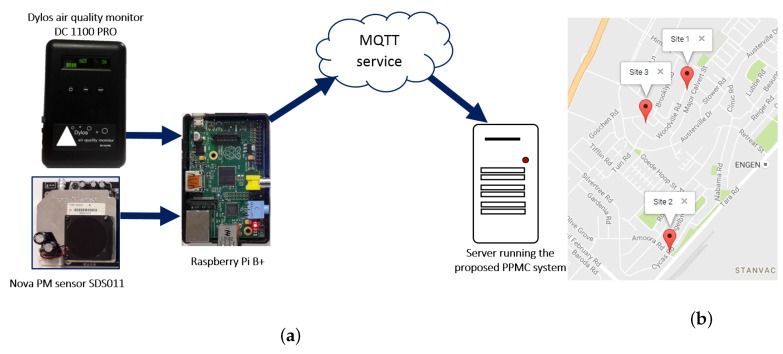
(**a**) Main hardware components; (**b**) Google map showing Site 1, Site 2 and Site 3.

Sensor units were installed in the three houses (Site 1, Site 2 and Site 3; See Figure 3). These were implemented with low-cost sensors, mounted on prototyping platforms such as Raspberry Pi to capture and format sensor observation data (PM${}_{2.5}$ concentration). The platforms also hosted communication devices to transmit the observation data to the processing server. The sensors sent streaming data over the Internet to the processing server located in the Cognitive and Adaptive Systems Research Laboratory, at the University of KwaZulu-Natal which is 20 km away. Site 1 is about 1.1 km away from Site 2, and about 300 m away from Site 3, while Site 2 and Site 3 are 900 m apart. The processing server hosts the knowledge base, and runs the monitoring and control system.

The hardware deployed in each site included a sensor network testbed implemented with low cost sensors for the monitored pollutants. PM${}_{2.5}$ was monitored with two different low cost sensors, Dylos air quality monitor DC1100 PRO (*Dylos monitor*) and Nova PM sensor SDS011 (*Nova sensor*). (http://www.dylosproducts.com/dc1100paqmc.html, http://inovafitness.com/en/Laser-PM2-5-Sensor-35.html) Using two low cost sensors for monitoring simultaneously allows for assuring the quality of the recorded observations. A Raspberry Pi B+ in each location acts as the sensor node that continually transmits streams of sensor observation data to the processing server. The sensor node is equipped with a LB-Link BL-WN151 wireless N adapter that connects to the Internet through HUAWEI E5330 mobile Wi-Fi router and transmits data to the server through a Message Queuing Telemetry Transport (MQTT) service.

The software for the PPMC includes the indoor environmental quality ontology reported on in an earlier study [1], which is now extended with terms to support prediction of future pollution levels and decision rules. The testbed was implemented with Apache Jena framework in Eclipse integrated development environment. C-SPARQL library, a stream reasoning engine and Apache Jena TDB, a triple store were also integrated into the framework.

A predictive model that employs a trained MLP, a Neural Network model to predict short term pollution levels of PM${}_{2.5}$, was implemented for the situation prediction component. This was implemented with the Waikato Environment for Knowledge Analysis (WEKA) [29] libraries in the Java environment and integrated in the architecture. The stream reasoning engine supports integrating both the current and future PM${}_{2.5}$ pollution states to determine appropriate feedback messages. An actuator module is then invoked to send pre-formatted control actions via SMS to the occupant when necessary.

### 3.3. Situation Prediction: Statistical Predictive Modeling

The situation prediction component of the PPMC system aims to predict short term trends of fine particulate matter in the indoor environment. Several factors have been noted to influence PM${}_{2.5}$ concentration in the indoor environment, such as indoor and outdoor sources of the particles, fine particles resting on different surfaces can also be resuspended in the air due to impact during activities. Activities, including sweeping, cooking, burning of incense and cigarette smoking are known to influence the concentration of PM${}_{2.5}$ captured in the sensor observation data. In this application scenario, a short time prediction is considered useful to take control of the impending unhealthy situation before it occurs.

A sensor data stream is essentially time series data, which requires a time series approach for predicting future values. Prediction of future states can be achieved by pattern *classification* with a *sliding window* technique [30,31]. Classification is an area of machine learning that involves constructing classifiers for characterizing datasets. A classifier is a function that maps the instances described by a set of attributes to one of a finite set of class labels [32]. Examples of classifiers include Bayesian Network classifiers, Neural Networks classifiers and Decision Trees classifiers. Classification techniques employ machine learning algorithms to identify and generate a model that fits the relationship between the attribute set and class label of the input data, such that the model can accurately predict class labels of new attribute sets [33]. Situation prediction in this application scenario is treated as *binary classification* [34]. The classifier is made to predict the PM${}_{2.5}$ state over a prediction horizon into one of two non-overlapping classes (*“Good”* or *“Poor”*) guided by the WHO recommended exposure limits for indoor PM${}_{2.5}$ [27].

The sliding window approach [30,31] for classification on time series data was adopted to predict PM${}_{2.5}$ short term pollution levels 30 minutes and 1 hour(h) into the future. A *sliding window* is a fixed length of data that slides through the temporally ordered data stream [30,32]. Sliding windows can be useful for two main purposes in time series data classification tasks. First, to select a fixed size of the most recent attributes from the evolving time series data as input for the classifier for predictions. Second, to slide through historical data and select a fixed size of data to update the classifier. In our approach, a sliding window is used to select attributes for generating feature-sets for the classifer to make predictions. Five different classifiers were considered for predicting PM${}_{2.5}$ short term pollution levels in this study. These are discussed below.

- *Bayesian Network (BN):* BN also referred to as *belief network* is an annotated directed acyclic graph that support representation of joint probability distribution over a set of random variables. A vertex in the graph represents a random variable while the edges represent dependencies between the variables. A conditional probability table is maintained at each node. A BN classifier can learn appropriate Bayesian network structure, and the probability tables from training data given the class variable. Classification is done based on joint probability distributions over class variables, given the particular instance of input variables. A class label with the highest posterior probability is predicted [32]. BayesNet is an implementation of BN in WEKA library [35].
- *Multilayer Perceptron (MLP):* MLP is one of a family of computation models called Artificial Neural Networks (ANN). They are used in machine learning and cognitive science to emulate the biological nervous system in computing functions. An ANN consists of several interconnected ’neurons’ and is capable of changing its structure based on the data that flows through it either from external or internal source. ANNs have been found notably suitable for non-linear classification tasks. MLP consists of three type of layers: the input layer, one or more hidden layers and the output layer. MLP has been widely and successfully used for time series prediction tasks [36].
- *Decision Table (DT):* DT is a rule based classifier which functions in the form of a look up table. DT consists of hierarchical tables such that each entry in a higher level table is broken down by the values of a pair of additional attributes to form another table, a process called decomposition [37]. As such, DT has two components, a list of attributes also called a schema, and a multiset of labeled instances referred to as the body. Every instance consists of a value for the label [38].
- *J48:* This is a Java implementation of the C4.5, a decision tree method. Decision tree classification methods build decision trees from labeled input datasets. A non-leaf node on the tree represents an attribute variable, while leaf nodes represents class variables. The J48 classifier implements a concept referred to as information gain, a mathematical tool which is used to measure the amount of information a dataset contains. This is used to assign the best fit variable in each of the nodes.
- *Random Forests (RF):* RF is an ensemble learning method. Ensembles are methods that implement several classifiers and aggregates their results. RF employs a method called bagging to aggregate results from several decision tree classifiers. Successive trees in bagging are independently constructed using a bootstrap sample of the dataset, such that a simple majority vote is taken on the result of the trees to make a prediction [39,40]. RF has been noted to give good performance on time series data [41,42].

#### 3.3.1. Data Pre-Processing and Feature Engineering

The data set consists of time series data of the historical PM${}_{2.5}$ concentration level generated from the sensor observation data. One week of continuous PM${}_{2.5}$ sensor observation data of one minute resolution was collected from each site for this study. The data was captured by two sensors, the Dylos monitor and the Nova sensor. Dylos monitor records PM${}_{2.5}$ observations in particle counts per cubic feet, while the Nova sensor records observation in micrograms per cubic meter (μg/m${}^{3}$). Conversion of the data from Dylos monitor to μg/m${}^{3}$ was achieved using the widely used method derived by Semple et al. [43,44].

Sensor data from low cost sensors can be inherently noisy. Hence, to minimize the noise in the data, a 30 min simple moving average of the actual 1 min resolution sensor observation data is used for the analysis. The sliding window technique maintains a queue of constant length in the form of *first in first out* (FIFO) with one minute resolution sensor observation data. At every minute a new sequence is formed which differs from the previous sequence only by addition of the newest time step observation data, and removal of the oldest time step observation data in the sequence. More formally, if $O_{t}$ represents the observation at current time *t*, at every time step, a new sequence consisting of a series of *n* observations is formed by pushing-in the new observation as $O_{t}$ and popping out the oldest observation $O_{t-(n-1)}$ from the previous sequence.

The features for building the classifiers include timestamps, mean of the sliding window sequence, class value for the mean, and class label for the target class. The class value and class label are categorical and binary, that is, two non overlapping classes (*“Good”* and *“Poor”*). Guided by the WHO recommended exposure limits to indoor PM${}_{2.5}$ [27], concentration values that are less than or equal to 25 μg/m${}^{3}$ are set to *“Good”* and those that are greater than 25 μg/m${}^{3}$ are set to *“Poor”* (see Table 1).

## 4. Experiments

Several experiments were carried out with time series techniques such as Auto Regressive Integrated Moving Average (ARIMA) but yielded no satisfying result for this case study. Hence, the adoption of a sliding window technique. 6480 data points of one minutes resolution, which corresponds to four and a half day continuous observation data was selected from each site data for analysis. The data was analyzed to select the appropriate machine learning algorithm for the case study and to determine the optimal training methods for the model. The experiments are described below:

### 4.1. Experiment 1: Data Visualization

The aim of the data visualization is to visualize the data from each site and understand class distribution of the data. First, the one minute resolution raw observation data from both Dylos monitor and Nova sensor were plotted together in line charts to show the trends of PM${}_{2.5}$ in the sites and also to see the agreement between the two sensors. Second, 30 min moving average data from both sensors was also plotted.

**Figure 4:**
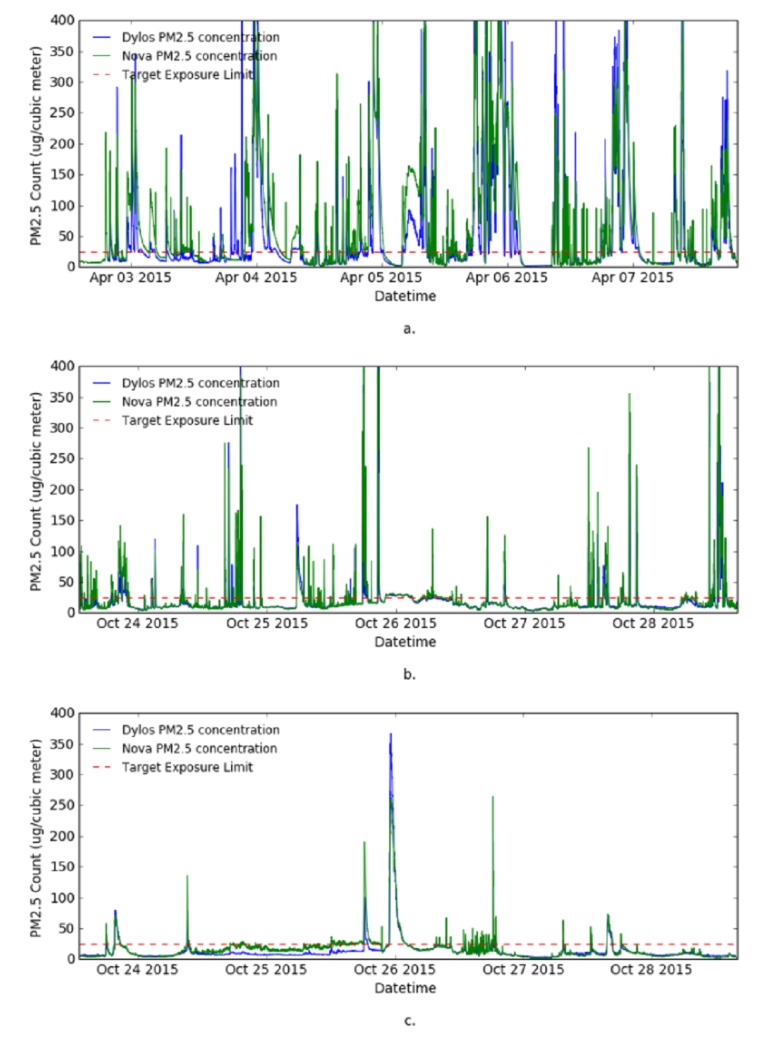
Line graph of raw sensor observation from the monitored sites. (**a**) Site 1; (**b**) Site 2; and (**c**) Site 3.

Figure 4 shows the visualization of the raw PM${}_{2.5}$ observations from the sites. The data captured by the Nova sensor (see Figure 4) is much more noisy compared to Dylos monitor observations (see Figure 4), therefore, data captured by the Dylos monitor is used for the remaining experiments. The figure shows Site 1 to be a heavily polluted house. This corresponds to the characteristics of the house; highly congested with one of the windows perpetually opened. Site 2 and Site 3 are much less polluted, they are cleaner and less congested. The high frequency of *“Poor”* class in Site 1 may also be due to seasonal variations, since Site 1 data was collected in April during the autumn season and data from the other two sites was collected in October during the spring season.

**Figure 5:**
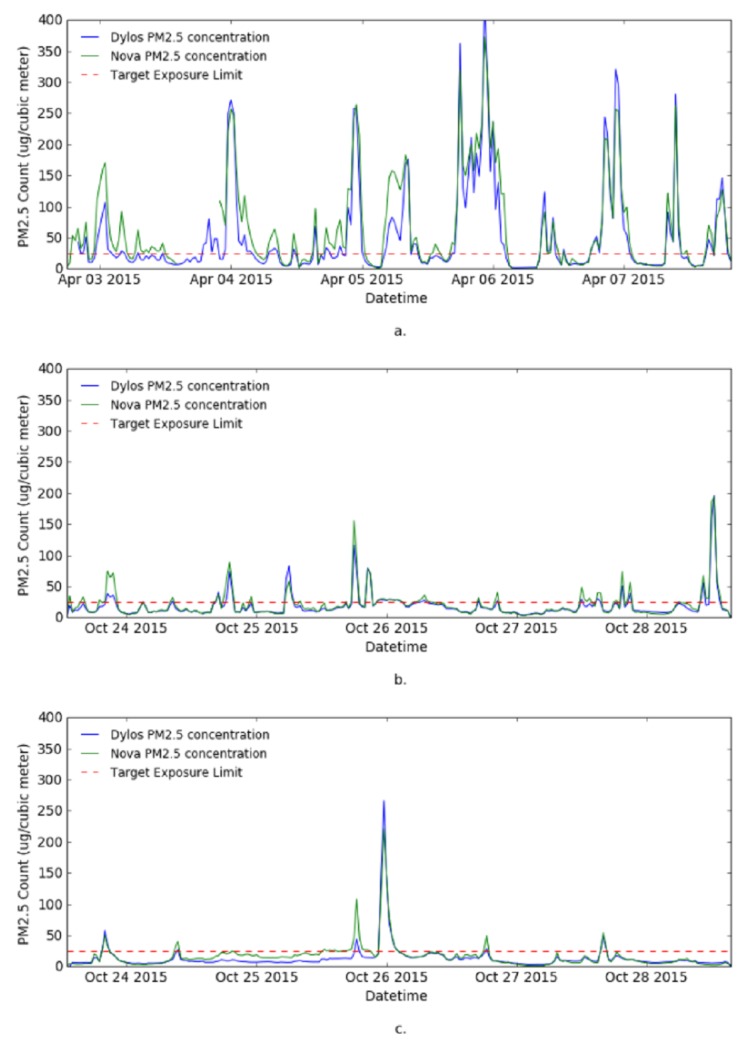
Line charts showing 1 min data from the monitored sites. (**a**) Site 1; (**b**) Site 2; and (**c**) Site 3.

Figure 5 shows the 30 min simple moving average of observation data from the three sites and the target exposure limit for PM${}_{2.5}$. From the graph, Site 1 is identified to fall in the category of the houses targeted for the PPMC system.

As a result of the visualization experiments, Site 1 is identified to fall into the category of the houses whose occupants are at risk of excessive exposure to fine particle pollution. Hence, the remaining experiments are performed on the data from Site 1, captured with Dylos monitor.

### 4.2. Experiment 2: Evaluation of Classifiers for Predictive Modeling

The aim of Experiment 2 is to select the appropriate classifier for a short term prediction of PM${}_{2.5}$ in the indoor environment. This experiment simulates the real live use case of the predictive model. For this experiment, the 1 min resolution data was further resampled to 30 min resolution such that a data point represents an average of sensor observation for the past 30 min. Resampling to 30 min resolution makes the prediction task over a 30 min horizon a one time-step prediction. The 30 min resolution data is used to generate input data for the classifiers in this experiment. The dataset is partitioned to allow for the classifiers to slide through the entire dataset at 6 h time-steps.

First, the model initializes by training the classifiers with the first 36 h observation data, then the classifier is made to predict target labels of unseen data for the following 6 h. After the prediction, the 6 h of unseen data is added to the training data and the classifier is retrained (see Table 2). This process is repeated through the entire dataset. All the classifiers were evaluated through the dataset in this manner. Table 2 shows the partitioning of the dataset for this experiment.

Two different classifiers were constructed and evaluated for each of the five different classification methods selected. The first classifier is trained to predict for the half hour horizon and the second classifier is trained to predict for one hour horizon.

#### 4.2.1. Evaluation Criteria

In order to evaluate the performance of selected classifiers, a *confusion matrix* (see Table 3) was constructed from the results of the classification, and the widely accepted metrics for binary classification tasks in machine learning community which include *Accuracy*, *Precision*, *Recall (Sensitivity)*, *Specificity* and *F-Measure* [34,45], were calculated from the confusion matrix. This classification task is focused on identifying the classifier that can better predict the *“Poor”* classes in the dataset over the prediction horizon. Therefore, when a *“Poor”* state is correctly classified as *“Poor”*, it is regarded as *true positive* (TP), and when a *“Good”* state is correctly classified as *“Good”*, it is regarded as *true negative* (TN). Likewise, a *“Good”* state wrongly classified as *“Poor”* is *false positive* (FP) and a *“Poor”* state wrongly classified as *“Good”* is *false negative* (FN). The counts of TP, TN, FP and FN predicted by the classifier is used to generate the confusion matrix (see Table 3) and the evaluation metrics as discussed below.

- *Accuracy:* Accuracy represents the overall performance of the classifier and it denotes the proportion of the whole testset (TP + FP + TN + FN) that are correctly classified (TP + TN) [34].
- *Precision:* Precision also referred to as *confidence* in Data mining community [45] denotes the proportion of predicted positive cases that are actually positive (*“Poor”*) in reality.
- *Sensitivity:* This is otherwise known as *recall* and it evaluates the proportion of the real positive states that are predicted positive [45].
- *Specificity:* Specificity or true negative rate is an inverse of recall, which denotes the proportion of real negative cases (*“Good”*) that are correctly predicted negative [34].
- *F-Measure:* F-Measure is an harmonic mean which combines precision and recall [34,45].

#### 4.2.2. Result

Table 4 presents the result of the evaluation on the classifiers for predictive modeling. Most of the classifiers show good precision and classification accuracy; however, for the analysis we are focused on not only precision but also on the balance between how sensitive the classifier is to the *“Poor”* states and how much it recognizes the *“Good”* classes (specificity). Random Forests classifier demonstrated the highest precision of 0.906 for the half hour prediction horizon but has the least sensitivity (0.774) (see Table 4). This is evident in the bias to the *“Good”* classes observed in the prediction task. The BN and the MLP demonstrate best performance in predicting PM${}_{2.5}$ states for the half hour horizon (see bold figures in Table 4), but the BN demonstrates lesser precision in predicting states for the one hour horizon. As a result of this experiment, MLP was chosen to model this case study.

### 4.3. Experiment 3: Evaluation of Sliding Window Sizes

This experiment aims to determine the optimal sliding window length for training the MLP that was selected for this study in Experiment 2. MLP classifiers were evaluated on four different datasets, each of which were prepared with different sliding window lengths (n=1, n=10, n=20 and n=30) and partitioned as shown in Table 2. The classifiers were made to predict next class values for both 30 min and 1 h prediction horizons. The performance of the classifiers in terms of precision, recall and specificity on each of set of the data was plotted in line charts.

#### 4.3.1. Result

**Figure 6:**
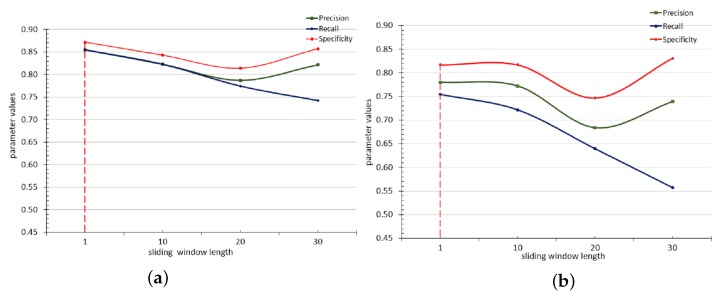
Line charts showing precision, recall and specificity against different sliding window length, (**a**) half hour prediction horizon; (**b**) one hour prediction horizon.

Figure 6 shows the result of this experiment. This experiment reveals that increasing the sliding window lengths of input data to the classifiers steadily decreases the performance of the classifiers in predicting the target classes. The point at which specificity and precision starts increasing when sensitivity (recall) keeps decreasing demonstrates a point where bias towards one of the target classes (*“Good”*) sets in, and starts increasing. That is, the model steadily loses sensitivity to the *“Poor”* class from this point. The dataset with window length n=1 gave the best performance (highlighted with dotted vertical lines in the Figure 6). Sensitivity especially demonstrates a free fall with the increase in sliding windows length. This observation may be due to the notion that more recent data is more relevant to the future than older ones [46]. A more detailed tabulated result of this experiment is presented in Table A1 of the appendix.

## 5. Integration of Predictive Model in the Framework

**Figure 7:**
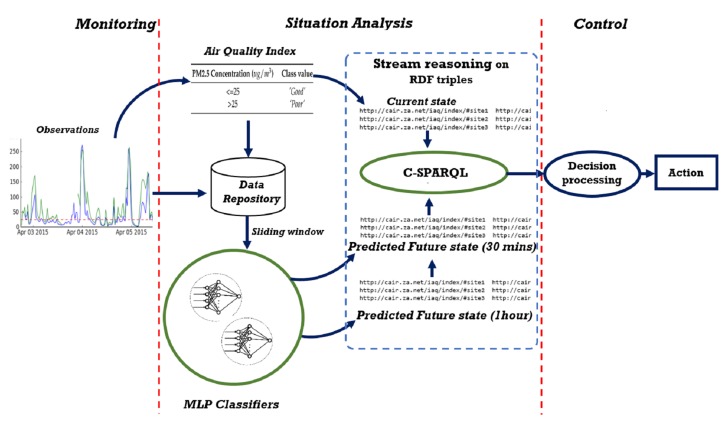
Integration of predictive modules into the PPMC system.

The selected MLP predictive classifier was integrated into the system using the WEKA library in Eclipse, a Java based Itegrated Development Environment. The situation prediction component consists of two different MLP classifiers to achieve two different horizons of prediction. The first was trained to predict pollution levels for the next half hour, and the second for the next one hour. The result of the situation prediction generated from the models is integrated into the stream reasoning framework by encoding it as Resource Description Framework (RDF) triples (see Figure 7). The C-SPARQL RDF stream reasoning engine supports registered queries to combine RDF streams and static RDF triples (in ontologies) for reasoning. Through this process, the RDF streams of predicted PM${}_{2.5}$ pollution trends which correspond to the future situation of the indoor air quality is combined with RDF streams of the current situation detected by the air quality index for decision processing.

### 5.1. The Monitoring and Control Process

**Figure 8:**
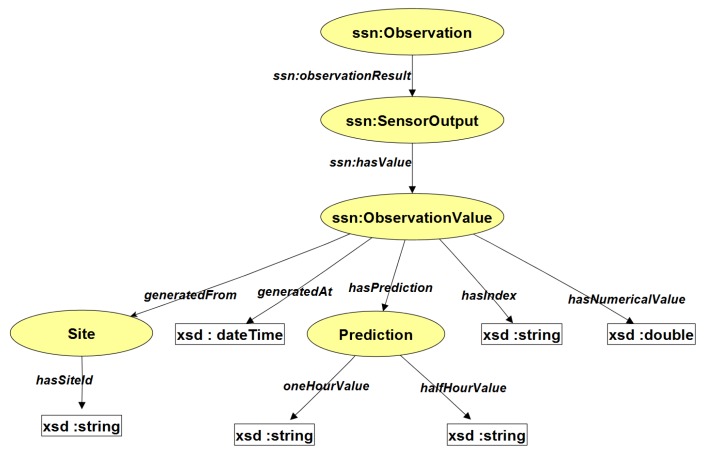
Fragment of the ontology showing the data model.

Three continuous queries are registered with the C-SPARQL engine to filter the RDF streams for air quality states at the current time (as indicated by the air quality index module) at the next half hour and at the next one hour. In order to be unobstructive, the system does nothing when the air quality is *“Good”*. At any time that either the current state or the predicted state is *“Poor”*, the decision processing module in the control layer is notified. The values detected by the monitoring queries are recorded in the ontology for reasoning by the decision processing module. Figure 8 shows a fragment of the ontology illustrating how an observation is stored. The model is based on the SSN ontology [8].

The following listings illustrate how triples are stored in the ontology, and how they can be processed for monitoring and control with continuous queries. We use *iaq-owl* as a shorthand notation for the Internationalized Resource Identifier (IRI).

- How Data is Stored in the ontology
  iaq-owl:SEQ2500 iaq-owl:generatedFrom iaq-owl:site01
  iaq-owl:SEQ2500 iaq-owl:generatedAt "01:25:12.100"^^xsd:**time**
  iaq-owl:SEQ2500 iaq-owl:hasPrediction iaq-owl:PRE7900
  iaq-owl:SEQ2500 iaq-owl:hasIndex "good"^^xsd:string
  iaq-owl:PRE7900 iaq-owl:halfHourValue "poor"^^xsd:string
  iaq-owl:PRE7900 iaq-owl:oneHourValue "poor"^^xsd:string
- *Monitoring Current Air Quality State*
  This query continually filters through the indoor air quality index stream to notify the decision manager of the current air quality detected by the index.
  **REGISTER QUERY** CurrentStateQuery
  **AS PREFIX** iaq-owl: <http://iaq-ukzn.ac.za/iaq.owl#>
  **SELECT** ?site ?current ?t
  **FROM STREAM** <http://iaq-ukzn.ac.za/iaqindex/**stream**> [RANGE 10 m STEP 10 m]
  **WHERE** {?seq iaq-owl:hasSeqID ?sid
      ?sid iaq-owl:generatedFrom ?site.
      ?sid iaq-owl:generatedAt ?t.
      ?pid iaq-owl:hasIndex ?current.
      **FILTER** (?current = "good"^^xsd:string)}
- Monitoring Half Hour Prediction State:
  This query monitors the predictions over 30 min horizon; it is activated to notify the decision manager when air quality predicted in the next 30 min is *“Poor”*.
  **REGISTER QUERY** halfHourPredictionQuery
  **AS PREFIX** iaq-owl: <http://iaq-ukzn.ac.za/iaq.owl#>
  **SELECT** ?site ?p1 ?t
  **FROM STREAM** <http://iaq-ukzn.ac.za/prediction/**stream**> [RANGE 10 m STEP 10 m]
  **WHERE** {?seq iaq-owl:hasSeqID ?sid
      ?seq iaq-owl:generatedFrom ?site.
      ?sid iaq-owl:generatedAt ?t.
      ?sid iaq-owl:hasPrediction ?p.
      ?p iaq-owl:halfHourValue ?p1.
      **FILTER** (?p1 = "poor"^^xsd:string)}
- Monitoring One Hour Prediction State
  This query is activated to notify the decision manager when air quality predicted in the next one hour is *“Poor”*.
  **REGISTER QUERY** oneHourPredictionQuery
  **AS PREFIX** iaq-owl: <http://iaq-ukzn.ac.za/iaq.owl#>
  **SELECT** ?site ?p2 ?t
  **FROM STREAM** <http://iaq-ukzn.ac.za/prediction/**stream**> [RANGE 10 m STEP 10 m]
  **WHERE** {?seq iaq-owl:hasSeqID ?sid
      ?seq iaq-owl:generatedFrom ?site.
      ?sid iaq-owl:generatedAt ?t.
      ?sid iaq-owl:hasPrediction ?p.
      ?p iaq-owl:oneHourValue ?p2.
      **FILTER** (?p2 = "poor"^^xsd:string)}

*RANGE* and *STEP* are operators used in C-SPARQL queries to support time windows. *RANGE* specifies the size of the time window that the query filters through, while *STEP* specifies time steps with which the time window slides forward. Setting both *RANGE* and *STEP* to the same value (for example 10 min as used in this use case) specifies a tumbling window scenario, in which the time window does not slide, but rather, at the end of a time window, another time window starts in a tumbling manner. This means that subsequent results do not contain observations from previous results. In this example, the window’s size is set to 10 min, but this can be set as desired.

The states values detected by the continuous queries can be used by the decision manager for reasoning with decision rules in the ontology in order to determine the appropriate actions at a point in time. For example, consider as a target future *“PM${}_{2.5}$ pollution”* situation a point when PM${}_{2.5}$ state is *“Poor”* consistently for up to thirty minutes. We can represent this in the system as when both the half hour and one hour prediction results are *“Poor”*, while the current state is not *“Poor”*. In this situation, the Proactive Pollution Monitoring and Control System needs to warn occupants to take some recommended proactive actions to avoid the predicted situation. The listing below demonstrates reasoning-logic by the decision manager in this example.

house(?site), sequence(?sid),
generatedFrom(?sid,?site),
hasIndex(?sid, !"poor"),
hasPrediction(?sid, ?pid),
halfHourValue(?pid, "poor")
oneHourValue(?pid, "poor"),
-> PM25pollutionPredicted(?site, ?**true**)

The decision rule can be implemented in any reasoning infrastructure that is compatible with the Semantic Web, such as Semantic Web Rule Language (SWRL), SPARQL or the JENA rule engine (www.w3.org/Submission/SWRL/, https://jena.apache.org/documentation/inference/#rules). In the use case scenario, when the pollution is predicted, the decision manager can activate the actuation module to send an appropriate control action to the occupants in order to prevent the pending unhealthy situations from happening. An example of this could be: “Alert: *Unhealthy Fine Particle Level predicted soon*; Proactive Control Advice: *Please avoid smoking, burning incense and excessive cooking indoors*”. More details about using activities to control indoor PM${}_{2.5}$ pollution is presented in our previous work [1].

## 6. System Analysis and Evaluation

In order to determine how the Proactive Pollution Monitoring and Control System will perform in the field, we carried out evaluation tests based on the test data used to evaluate the classifiers (see Section 4). The test data consists of 132 observations in all. The data was made to run through the components of the system. The performance of the components and the overall efficiency of the system was analyzed. The system used for the evaluation is an ASUS laptop running Windows 7, with Corei5 (Intel(R) Core(TM)i5-3337U CPU @1.80GHz) processor and 12.0 GB installed memory. Result of the analysis and evaluation of the system with respect to design decisions made on each of the components are discussed below.

The situation prediction component initializes by training the classifiers with 36 h of historical data (see Section 4.2). Over ten runs, the average initialization time was 39,208.0 ms (≈0.65 min) to train MLP classifiers for the half hour prediction and 47,098.4 ms (≈0.78 min) to train the classifiers for the one hour prediction. The classifiers then effectively processed each subsequent prediction task in a maximum of 1 ms in all the cases. However, the system is also designed to update the classifiers every 6 h with the most recent data. We compared the training times of the MLP classifiers with that of BN classifiers which was found equally suitable for this work (see Section 4.2). Table 5 shows the variation of training time as the size of datasets grows. The re-training time for MLP classifiers increases rapidly as the dataset grows, while the re-training time of BN is minimal and remains relatively constant after the initialization. This experiment reveals that although the MLP model has a slightly better predictive performance than BN in this study, it is not as scalable as BN. Hence, the choice of MLP over BN for the system is a trade-off between the predictive performance and scalability. Given the poor model update speed of the MLP as the data set grows, the BN is a more likely choice for implementation. However, further investigation is required on mechanisms for reducing the model update time for the MLP.

The situation detection component, which detects the current situation by interpreting observation data based on the air quality index (see Section 3.3.1) identifies all the situations correctly. The output of this component also serves directly as labeled data for retraining the classifiers during system updates.

Stream reasoning with C-SPARQL is used to monitor three different streams (see Section 5.1) in the system, that is, the current pollution situation, the half hour prediction and the one hour predictions. Out of the 132 observations in the test data, 62 observations have either half hour predictions or one hour predictions that are “Poor”. The queries effectively detected all the targeted situations correctly. Detected situations are appropriately recorded in the ontology.

The decision to activate alarms is based on the result of a SPARQL query that is evaluated on the ontology at specified intervals, which was set to 10 min for the purpose of this evaluation. The query filters through the data to detect situations in which half hour and one hour predictions are both “Poor” for the past 10 min in order to activate control actions. When C-SPARQL is used to filter the predictions, only 62 prediction triples that have either half hour prediction or one hour predictions as “Poor” were recorded in the ontology. In a query test that was repeated ten times, the average execution time of SPARQL query was found to be 295 ms. We compared this with the execution time of SPARQL query when all the observations were streamed into the ontology, that is, when C-SPARQL is not used. The dataset in the ontology now includes the triples representing the predictions of all the 132 observations. The average execution time of the query is 441 ms. The difference of 146 ms may seems little because of the minimal dataset for now, but as the number of triples in the ontology grows, the performance difference may be much more pronounced. Stream reasoning queries could also have been used to activate decisions on the fly, without storing data in the ontology, however, the ontology supports combining the stream reasoning with other static data pre-captured in the ontology including the control actions to be recommended to the occupants.

In order to asses the overall effectiveness of the system, we compared the number of times that the system raised alarms for predicting pollution with the number of times that the corresponding records in the actual data specifies that both half hour situations and one hour situations are “Poor”. Out of the 132 observations in the test data, the “Poor” condition is satisfied 52 times, however, the system raised alarms 59 times, giving 7 (11.86%) false alarms. The false alarms were found to be due to false positive predictions by the situation prediction component.

## 7. Related Work

There is growing research interest in the design and application of proactive systems. Some of the proactive techniques that have been proposed have been reviewed by VanSyckel et al. [47]. Wang and Cao [48] reported on a proactive method for large-scale transportation Internet of Things. They integrated a multi-layered Adaptive Dynamic Bayesian Network predictive method into a probabilistic event detection system and applied it to predict and abate traffic congestion in a simulated environment. This study is different from Wang and Cao’s [48] in that it integrates statistical machine learning into Semantic Sensor Web applications using stream reasoning techniques. Wang and Cao did not utilize semantic sensor techniques nor did they apply stream reasoning methods.

Anaya [23] integrated predictive analysis in self-adaptive systems. The author proposed statistical machine learning techniques for predicting the future, and fuzzy logic for control mechanisms. Although the concept of this work is similar to Anaya’s [23], who sought to achieve proactive behaviors by integrating predictive analysis in a context aware system, he did not utilize semantic methods.

There has also been previous work done on air quality monitoring and control. Yu et al. [20] proposed an intelligent wireless sensing and control system to improve indoor air quality: monitoring, prediction, and pre-action. They used low cost sensors for monitoring indoor carbon dioxide (CO${}_{2}$) levels and employed the integration of Auto Regression Moving Average (ARIMA) time series forecasting method to predict future concentration levels of CO${}_{2}$ and they used fuzzy logic to make control decisions. This study is different from Yu et al. [20] in many ways. First, they based air quality on CO${}_{2}$ concentration levels, while this study is focused on air quality with respect to fine particulate matter (PM${}_{2.5}$) concentration levels. Second, the uncertainty in the continuity of indoor levels of PM${}_{2.5}$ requires techniques different from ARIMA to predict future levels. We employed a sliding window classification approach to predict PM${}_{2.5}$ pollution levels. Finally, this study advocates Semantic Sensor Web techniques to manage semantically annotated streaming observation data, which was not used by Yu et al.

Saad et al. [21] proposed Artificial Neural networks in indoor air quality monitoring system. Their work is related to this study in two ways. First their study was based on indoor air quality monitoring, and second they proposed the use of a Neural Network model for pattern recognition. However, their work is different from this study in the following ways. Firstly, their application of the Neural Network model was to identify the sources of indoor air pollution and not to predict future trends like ours. Secondly, their system was not focused on proactive control like ours, and thirdly, they did not employ semantic techniques in their study.

Artificial Neural Network models have been found useful for short term prediction tasks in many intelligent real time application scenarios. Dia [49] successfully employed Neural Network for predicting short time traffic situations from 5 to 15 min into the future with great accuracy. Kani and Ardehali [50] also proposed a Neural Network model for predicting short term wind speed few seconds, minutes and about an hour into the future. In this work, MLP, a Neural Network classifier also proves useful in predicting short term trend of PM${}_{2.5}$ 30 min and 1 h into the future with great precision and sensitivity.

Vafaeipour et al. [30] reported on a successful sliding window approach with neural networks models on time series data to predict wind velocity. Their work is related to this study as regards using sliding window with Neural Network model on time series data. However, their work is different from ours in the following ways. First, their approach was focused towards regression, while the approach used in this study is directed towards binary classification. Second, their application was in an entirely different domain; they are predicting ambient wind speed for power generation, while the predictive model in this study is applied to PM${}_{2.5}$ trends in the indoor environment.

The high precision of up to 0.86 (86%) achieved in predicting trends of indoor PM${}_{2.5}$ in this study is comparable with Yu et al. [51] achieved a precision of 81% in inferring ambient air quality index with a Random Forests classification approach, especially PM${}_{2.5}$ in urban areas. While their approach was based on using several attributes from urban sensing systems, this study employs solely time series observation data from low cost sensors and a Neural Network model. This study suggests that fine particle pollution can be predicted with comparable high precision or perhaps better in the indoor environment by applying Neural Networks classifiers on data from low cost sensors.

## 8. Discussion and Conclusions

We have presented an approach to achieve proactive monitoring and control in the Semantic Sensor Web by integrating a statistical prediction model in the processing space of a stream reasoning framework. The proactive monitoring and control approach was illustrated with an indoor air quality scenario and data streams from a real live case study in a low-cost residential setting. The proposed system provides a mechanism to combine the high accuracy and performance of statistical predictive techniques and the expressiveness of semantic analytic techniques for proactive monitoring and control. Although the concept of proactive computing is not new [22,23], many Semantic Sensor Web monitoring applications are still designed in reactive manners. The reason is perhaps due to the fact that the predictive methods, such as predictive reasoning [17,18], that are native to Semantic Web technologies, are still emerging [10]. And although recent works in the stream reasoning community offer support for integration of heterogeneous data stream sources, more work is needed, especially on the approaches to integrate predictive models within the processing space of a stream reasoning framework for Semantic Sensor Web applications. This study proposes an architecture that attempts to fill this gap. The architecture was shown to be effective for combining both stream reasoning processes and the outputs of predictive models for predicting situations of interest. While the mechanism was designed to be application interdependent, we are in the process of testing it on other applications to verify this.

Secondly, we propose a sliding window approach that employs MLP classifiers for predicting states of indoor PM${}_{2.5}$ pollution levels from low cost sensor observation data streams. This study suggests that predicting particulate matter pollution levels in the indoor environment requires a different technique than ARIMA proposed by Yu et al. [20] for indoor air quality based on CO${}_{2}$. The unsatisfactory results observed with ARIMA may be due to the uncertainty in the continuity of the PM${}_{2.5}$ levels in the indoor environment. From the dataset used for this study, the concentration level of indoor PM${}_{2.5}$ does not follow a regular pattern. Hence, it may be difficult for a method like ARIMA to effectively predict indoor levels of PM${}_{2.5}$ in this study.

The sliding window approach to manage time series data for prediction has been demonstrated to be useful for modeling other real time environmental domains [30,31]. This study demonstrates that the sliding window approach can also be used with a MLP model on time series data to predict indoor PM${}_{2.5}$ pollution levels with high precision and sensitivity. As far as we know, our work is the first attempt to use this approach for predicting PM${}_{2.5}$ in the indoor environment. And although some efforts have employed Dylos sensors for detection of particulate matter, to the best our knowledge, our work is the first approach to attempt predicting future levels of PM${}_{2.5}$ with data from Dylos 1100 PRO, a low cost particle sensor.

In real time data stream scenarios, especially in a dynamic environment, the relationship between the data and the properties of the target variable, which the statistical models predict is known to drift over time (*concept drift*) [52]. Sliding window methods are among approaches that have been proposed to overcome concept drifts [53].

This study further suggests that an appropriately trained MLP and BN classifiers can effectively predict short term trend of PM${}_{2.5}$ with high precision and sensitivity. In our experiments, we achieved precision of up to 0.86 and sensitivity of up to 0.85 using these two classifiers with our sliding windows approach for predicting PM${}_{2.5}$ states 30 min into the future.

MLP, a member of the Artificial Neural Network family of models and BN prove to be the best method for modeling the situation prediction component of the proactive monitoring and control framework, given the case study at hand. Although MLP demonstrated a slightly better performance, it is known to be difficult to train [54], making the choice between MLP and BN a trade-off between predictive performance and scalability. This suggests that a particular method may not be applicable in all scenarios. Therefore, careful experiments should be carried out to determine the best predictive model for each specific situation to be modeled.

The use of stream reasoning in this study fulfills three important design goals. First, it provides a means to integrate the output of situation prediction into the system for further processing. Secondly, it ensures that only target situations are recorded in the ontology, which is in turn important for better query performance on the ontology. And finally, stream reasoning is an elegant means to keep the system’s components decoupled, which supports easy reconfiguration without the need for hardwiring of the components. However, existing stream reasoning approaches still have known limitations with respect to lack of standards, performance issues and the maturity of reasoning support [55]. More expressive queries take a longer time to execute, especially when applied to large real time data streams. We expect that ongoing efforts in the stream reasoning community are addressing these potential shortcomings [56] (https://www.w3.org/community/rsp/).

The combination of Machine Learning and ontology driven components in an architecture as demonstrated in this study, especially the use of the data output of the situation detection as labeled data for retraining classifiers for situation prediction, highlights an added value. This suggests that semantic components of the system can support and enhance the functionalities of the predictive components. This in turn may enhance the dynamism of the system and improve automation.

The decision processing of the proactive system is demonstrated by reasoning on the ontology in Section 5 and Section 6. In an ongoing effort, we are investigating advanced proactive decision processing mechanisms, which incorporate the classical principles of decision theory for Semantic Sensor Web applications. We are also interested in investigating how ontologies can capture the pattern of predictive errors made by the system, which may be useful in minimizing the false alarms raised.

## Figures and Tables

**Table 1:** Class values guided by WHO recommended exposure limits for indoor PM${}_{2.5}$ [27].

PM${}_{2.5}$ Concentration (μg/m${}^{3}$)	Class Value
≤25	*“Good”*
>25	*“Poor”*

**Table 2:** Dataset partitions for evaluating classifiers.

Training		Train Set Size	Testing		Test Set Size
From	To		From	To	
3 April 2015 10:00	4 April 2015 21:30	72	4 April 2015 22:00	5 April 2015 3:30	12
3 April 2015 10:00	5 April 2015 3:30	84	5 April 2015 4:00	5 April 2015 9:30	12
3 April 2015 10:00	5 April 2015 9:30	96	5 April 2015 10:00	5 April 2015 15:30	12
3 April 2015 10:00	5 April 2015 15:30	108	5 April 2015 16:00	5 April 2015 21:30	12
3 April 2015 10:00	5 April 2015 21:30	120	5 April 2015 22:00	6 April 2015 3:30	12
3 April 2015 10:00	6 April 2015 3:30	132	6 April 2015 4:00	6 April 2015 9:30	12
3 April 2015 10:00	6 April 2015 9:30	144	6 April 2015 10:00	6 April 2015 15:30	12
3 April 2015 10:00	6 April 2015 15:30	156	6 April 2015 16:00	6 April 2015 21:30	12
3 April 2015 10:00	6 April 2015 21:30	168	6 April 2015 22:00	7 April 2015 3:30	12
3 April 2015 10:00	7 April 2015 3:30	180	7 April 2015 4:00	7 April 2015 9:30	12
3 April 2015 10:00	7 April 2015 9:30	192	7 April 2015 10:00	7 April 2015 15:30	12

**Table 3:** Confusion matrix.

Actual Class Value	Classified as *“Poor”*	Classified as *“Good”*
*“Poor”*	TP	FN
*“Good”*	FP	TN

**Table 4:** Precision, sensitivity, specificity and F-Measure of evaluated classifiers on Site 1 dataset.

Prediction Horizon	Classifier	Accuracy	Precision	Sensitivity	Specificity	F-Measure
	BN	0.864	**0.855**	**0.855**	**0.871**	0.855
	DT	0.856	0.864	0.823	0.886	0.843
30 min	J48	0.856	0.852	0.839	0.871	0.846
	MLP	0.864	**0.855**	**0.855**	**0.871**	0.855
	RF	0.856	0.906	0.774	0.929	0.835
	BN	0.780	**0.758**	**0.770**	**0.789**	0.764
	DT	0.773	0.804	0.672	0.859	0.732
1 h	J48	0.773	0.816	0.656	0.873	0.727
	MLP	0.788	**0.780**	**0.754**	**0.817**	0.767
	RF	0.758	0.822	0.607	0.887	0.698

**Table 5:** Performance of situation prediction classifiers during updates.

Dataset Size	MLP		BN	
	One Hour Classifier	Half Hour Classifier	One Hour Classifier	Half Hour Classifier
	Training Time (ms)	Training Time (ms)	Training Time (ms)	Training Time (ms)
72	39,208.0	47,098.4	288.2	301.2
84	45,280.4	49,701.2	4.8	3.7
96	54,979.6	55,967.2	4.2	3.0
108	64,487.0	62,518.6	3.4	3.7
120	74,083.8	65,513.8	3.4	3.1
132	76,995.2	71,036.4	3.2	2.9
144	85,660.4	81,994.4	3.0	3.5
156	92,779.4	91,990.8	3.2	4.1
168	92,376.6	97,852.6	4.2	7.0
180	97,573.0	104,304.6	3.8	3.3
192	109,404.8	111,131.4	4.8	4.5

## Abbreviations

The following abbreviations are used in this manuscript:

SSW	Semantic Sensor Web
PM${}_{2.5}$	Fine particles of aerodynamic diameter less than 2.5 µm
PPMC	Proactive Pollution Monitoring and Control System
BN	BayesNet
MLP	Multilayer Perceptron
DT	Decision Table
RF	Random Forests
RDF	Resource Description Framework

## Appendix A

**Table A1:** Accuracy, precision, recall, specificity and F-measure of MLP classifiers in dataset with different sliding windows.

	Sliding Window Length	Accuracy	Precision	Recall	Specificity	F-Measure
	1	0.864	0.855	0.855	0.871	0.855
30 min	10	0.833	0.823	0.823	0.843	0.823
	20	0.795	0.787	0.774	0.814	0.780
	30	0.803	0.821	0.742	0.857	0.780
	1	0.788	0.780	0.754	0.817	0.767
1 h	10	0.773	0.772	0.721	0.817	0.746
	20	0.697	0.684	0.639	0.746	0.661
	30	0.705	0.739	0.557	0.831	0.636
